# Endotoxins and Non-Alcoholic Fatty Liver Disease

**DOI:** 10.3389/fendo.2021.770986

**Published:** 2021-10-29

**Authors:** Takaomi Kessoku, Takashi Kobayashi, Kento Imajo, Kosuke Tanaka, Atsushi Yamamoto, Kota Takahashi, Yuki Kasai, Anna Ozaki, Michihiro Iwaki, Asako Nogami, Yasushi Honda, Yuji Ogawa, Shingo Kato, Takuma Higurashi, Kunihiro Hosono, Masato Yoneda, Takayuki Okamoto, Haruki Usuda, Koichiro Wada, Noritoshi Kobayashi, Satoru Saito, Atsushi Nakajima

**Affiliations:** ^1^Department of Gastroenterology and Hepatology, Yokohama City University Graduate School of Medicine, Yokohama, Japan; ^2^Department of Palliative Medicine, Yokohama City University Hospital, Yokohama, Japan; ^3^Department of Pharmacology, Shimane University Faculty of Medicine, Izumo, Japan; ^4^Department of Oncology, Yokohama City University Hospital, Yokohama, Japan

**Keywords:** NAFLD, leaky gut, endotoxin, intestinal permeability, small intestinal bacterial overgrowth

## Abstract

Nonalcoholic fatty liver disease (NAFLD) is the most common chronic liver disease worldwide. It occurs with a prevalence of up to 25%, of which 10–20% cases progress to nonalcoholic steatohepatitis (NASH), cirrhosis, and liver cancer. The histopathology of NASH is characterized by neutrophilic infiltration, and endotoxins from gram-negative rods have been postulated as a contributing factor. Elevations in endotoxin levels in the blood can be classified as intestinal and hepatic factors. In recent years, leaky gut syndrome, which is characterized by impaired intestinal barrier function, has become a significant issue. A leaky gut may prompt intestinal bacteria dysbiosis and increase the amount of endotoxin that enters the liver from the portal vein. These contribute to persistent chronic inflammation and progressive liver damage. In addition, hepatic factors suggest that liver damage can be induced by low-dose endotoxins, which does not occur in healthy individuals. In particular, increased expression of CD14, an endotoxin co-receptor in the liver, may result in leptin-induced endotoxin hyper-responsiveness in obese individuals. Thus, elevated blood endotoxin levels contribute to the progression of NASH. The current therapeutic targets for NASH treat steatosis and liver inflammation and fibrosis. While many clinical trials are underway, no studies have been performed on therapeutic agents that target the intestinal barrier. Recently, a randomized placebo-controlled trial examined the role of the intestinal barrier in patients with NAFLD. To our knowledge, this study was the first of its kind and study suggested that the intestinal barrier may be a novel target in the future treatment of NAFLD.

## 1 Introduction

Non-alcoholic fatty liver disease (NAFLD) is the hepatic manifestation of metabolic syndrome and leading cause of chronic liver disease in pediatric and adult populations living in industrialized countries. NAFLD encompasses steatosis and non-alcoholic steatohepatitis (NASH) and is characterized by periportal and lobular inflammation. Progression to fibrosis and cirrhosis are the primary complications of NAFLD (1). Based on a recent meta-analysis, one in four people in Europe, the United States, and Asia have NAFLD (2). The “multiple-hit” hypothesis may explain the pathogenesis and progression of NAFLD. In recent years, there has been increasing interest in gut-liver axis dysfunction, which is characterized by dysbiosis, bacterial overgrowth, and changes in intestinal permeability. Gut-liver axis dysfunction is considered the second hit that results in the progression of NAFLD. As such, gut-liver axis dysfunction is considered as an important alternative therapeutic target for patients who do not benefit from lifestyle changes, healthy eating, and physical activity (3, 4). The first hit behind the chronic inflammation in NASH is triggered by fat accumulation in the hepatocytes, followed by exposure to inflammatory cytokines, insulin resistance, oxidative stress, lipotoxicity, mainly from free fatty acids (as), and gut-derived endotoxins. Here, we focused on gut-derived endotoxins and reviewed the most recent data regarding the gut-liver axis and its role in the pathogenesis and progression of NAFLD.

## 2 Endotoxins and NAFLD

Fatty liver is caused by excessive caloric consumption from overeating, obesity, and lack of exercise. While fatty liver commonly develops from exposure to inflammatory cytokines, insulin resistance (IR), oxidative stress, lipotoxicity (mainly from FFAs), genetic predisposition, and exposure to intestinal bacterial endotoxins also play a role in its pathogenesis.

Patients with NASH have high levels of endotoxins in their blood (5). In recent years, metabolic endotoxemia, which is defined as an increase in serum endotoxin levels in response to a high-fat Western diet, has gained increasing recognition (6). Among the intestinal microflora, gram-negative bacilli are considered the largest source of stable endotoxins. The primary endotoxin produced by gram-negative bacilli include lipopolysaccharides (LPS). As the intestinal environment deteriorates, anaerobic gram-negative rods proliferate, and the amount of LPS produced by these organisms also increases. While the elaborate immune system and functional intestinal barrier prevent all these endotoxins from entering the portal vein, it is likely that some still reach the hepatic vault. When intestinal enterobacteria invade the portal vein, the first target organ is the hepatic vasculature. This suggests that Enterobacteriaceae-derived endotoxins are important in the inflammatory response that leads to the development of NASH. Endotoxins are pathogen-associated molecular patterns (PAMPs), which are members of the toll-like (TLR) and nucleotide-binding oligomerization domain (NLRs) groups of receptors.

**Figure 1:**
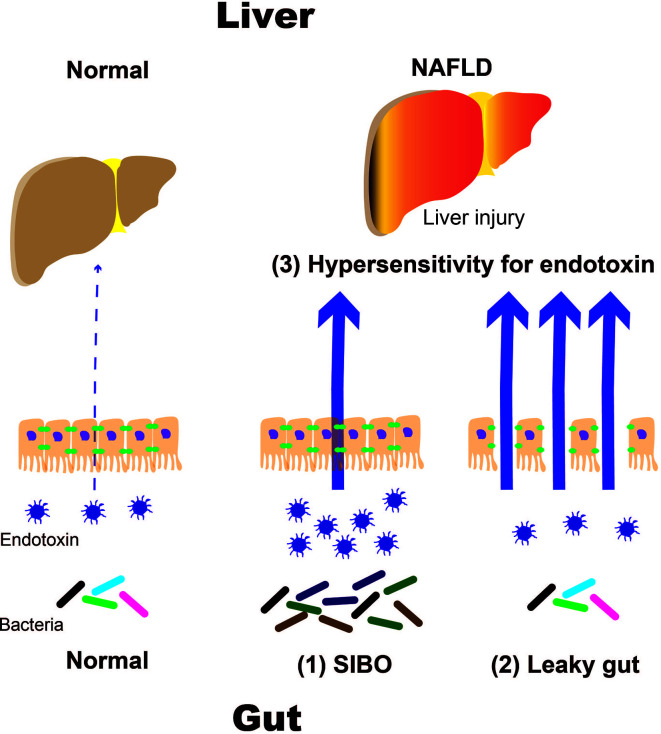
Mechanisms of NAFLD progression caused by intestinal and hepatic factors. NALFD, non-alcoholic fatty liver disease; SIBO, small intestinal bacterial overgrowth.

In particular, TLR4 is expressed in the plasma membrane of hepatocytes and Kupffer cells. Endotoxins stimulate TLR4, which activates signaling molecules, such as nuclear factor kappa B (NF-κB). This leads to the production of inflammatory cytokines, namely interleukin (IL)-1β and IL-18, which results in liver injury. Downstream targets of TLR4 signaling are determined by selective recruitment of cytosolic sorting and signaling adaptor proteins *via* interactions between Toll/IL-1 receptor (TIR) domains (7–9). Thus, TLR4 activation may engage myeloid differentiation factor 88 (MyD88) and TIR domain-containing adaptor protein or MyD88 adaptor-like factors, leading to the activation of nuclear factor kappa-B (NF-κB) and activator protein 1 transcription factors (10–12). There is substantial evidence that TLR4-mediated cellular events escalate liver injury in NAFLD (10, 12). Recent studies indicated that TLR4 sorting specificity might reflect the etiology of fatty liver disease. Due to the ubiquitous presence of TLR4 among various types of liver cells, the specific role of Kupffer cells in differential activation of TLR4 pathways remains to be determined. It must also be noted that endogenous ligands such as certain FFA and other alarmins may also be linked to TLR4 sorting specificity, a question particularly relevant to NAFLD. Antimicrobial therapy reduces hepatic damage in patients with NASH. Animal studies have shown that TLR4-deficient mice did not develop NASH, which suggests that intestinal bacteria play an important role in the emergence of NASH. Intestinal bacteria have been proposed to contribute to the development of NASH through several mechanisms. Intestinal factors include (1) disruption of the intestinal barrier function, which results in a leaky gut and (2) qualitative and quantitative dysregulation of intestinal bacteria, such as in short intestinal bacterial overgrowth (SIBO). Increased responsiveness to endotoxins has been identified as a hepatic factor (**Figure 1**).

### 2.1 Measurement of Blood Endotoxins

Unlike pathologic conditions like sepsis, NAFLD has insignificant amounts of endotoxins. We utilized the HK302 (HyCult Biotechnology, Uden, The Netherlands) and HK503 (HyCult Biotechnology) enzyme-linked immunosorbent assay (ELISA) kits to detect trace amounts of endotoxins and lipopolysaccharide-binding proteins (LBP), respectively, in the portal vein. In humans, blood endotoxins were measured with an endotoxin activity assay (Toray Medical Co., Ltd., Tokyo, Japan) (13, 14) or LBP levels (HK315 ELISA kit; HyCult Biotechnology) (15). LBP levels can be utilized to measure endotoxin levels with commercially available limulus amebocyte lysate chromogenic endpoint assays (Hycult Biotech), which have concentrations that range from 0.04–10 EU/mL. Whole blood endotoxin activity assays (EAAs) were performed as described previously (16–18) with a murine immunoglobulin (Ig)M monoclonal antibody against lipid A of *Escherichia coli* J5. Whole blood samples (40 μL) were incubated in duplicate with saturating concentrations of antibody and stimulated with zymosan. The resulting respiratory burst activity was detected as light released from the lumiphore luminol and measured with a chemiluminometer (Toray Medical Co., Ltd.). EAAs have been used in recent NAFLD clinical trials (19).

## 3 Leaky Gut Syndrome

When the contents of the gastrointestinal tract cross the intestinal wall, they pass through tight junctions in the epithelial space and are met by immune surveillance cells. Depending on the invading cells, the immune surveillance cells secrete the inhibitory cytokine IL-13 to synthesize additional tight junctions to strengthen the interepithelial barrier. When the invading cells are pathogenic microorganisms, the surveillance cells respond with inflammatory reactions. Cytokines can destroy tight junctions on their own. As a result, cytokines can invade the intestinal tract and white blood cells. Cytokines can also mobilize immune cells to the mucosal surface to fight invading microorganisms. As such, submucosal immune surveillance cells function as the control tower that dynamically changes tight junction activity to provide a biologic defense. Increased intestinal permeability and intestinal microbiota were first proposed as the possible etiologies behind some diseases in 1890 (20). The crosstalk between the gut and liver is an interesting hypothesis that can explain the changes in the hepatobiliary systems of patients with inflammatory bowel disorders, such as celiac disease, and infectious bowel conditions caused by *Salmonella* and *Yersinia (*21). Recent evidence has demonstrated that the hepatointestinal system may be involved in the development of NASH (22–24).

Obesity increases intestinal permeability by indirectly damaging the intestinal barrier (24–26). A high-fat diet (HFD) may increase the risk for metabolic endotoxemia and reduce intestinal *Bifidobacteria* (27, 28). *Bifidobacteria* lower intestinal LPS levels and improve mucosal barrier function (25). Mechanisms that regulate intestinal barrier health may also regulate the degree of endotoxemia (24–26, 29). Instead of disrupting tight junctions between cells, increased LPS absorption in the intestine may also occur secondary to chylomicron secretion from the enterocytes. Cell culture and animal model studies have suggested that endotoxins are actively secreted into the blood along with chylomicrons. Inhibition of chylomicron synthesis has been demonstrated to inhibit endotoxin secretion (31). These data suggest that gut microbiota-derived endotoxins are strongly associated with the development of NASH through impaired intestinal barriers and increased chylomicron secretion by enterocytes.

In recent years, patients have demonstrated very slight decreases in barrier function in the absence of subjective symptoms; this condition is referred to as leaky gut syndrome. Fatty diets, such as Western-style diets, reportedly reduce the intestinal barrier function, which increases serum endotoxin levels (6). The endotoxins in this study were derived from gram-negative bacilli, which suggested that they were derived from the gut microbiota of the intestinal tract. When patients were shifted to a normal diet, serum endotoxin levels normalized, which indicated that changes in dietary content may affect intestinal barrier function.

Leaky gut syndrome is characterized by decreased intestinal barrier function and its pathologic sequelae. The etiology of leaky gut syndrome is quite diverse and may include high-fat and fructose diets, drugs, and age. Several observational studies and animal experiments have also demonstrated that leaky gut syndrome is associated with a wide variety of diseases. It is speculated that various molecules enter the bloodstream through a leaky gut. Among these molecules, the most significant seems to be endotoxins. When endotoxins enter the bloodstream, even minute amounts may cause sepsis and death. Minute amounts of endotoxins have also been documented to cause liver damage because the liver is highly sensitive to endotoxins. Endotoxins have also been associated with IR in adipose tissue and arteriosclerosis of the arterial wall. Given the above data, a leaky gut allows minute amounts of endotoxins into the bloodstream, which results in chronic inflammation. Chronic inflammation is the basis of the pathophysiology of lifestyle-related diseases, such as diabetes, arteriosclerosis, and chronic kidney disease. It is thought to play an important role in high-grade inflammation, which is also known as metabolic endotoxemia.

### 3.1 Leaky Gut Syndrome and NAFLD

NAFLD and NASH are the most common liver diseases associated with Westernized eating habits. Westernized eating habits impair the intestinal environment, which results in anaerobic gram-negative rod proliferation. In particular, obese patients with NASH who consume high-fat and fructose diets have increased intestinal permeability. These may result in an influx of endotoxins that can travel up to the liver *via* the portal vein. Endotoxins react with receptor groups, such as TLRs and NLRs, as PAMPs. TLR 4 is expressed on the cell membranes of hepatocytes, Kupffer cells, and other similar cells. TLR 4 mediates the activation of signaling molecules, such as NF-κB, which activate inflammatory cytokines like IL-1β. It is thought that IL-18 is produced following liver damage. (1) Increased intestinal permeability, such as in leaky gut syndrome and (2) qualitative and quantitative abnormalities in intestinal bacteria, such as in SIBO, have been proposed to play important roles in the pathogenesis and progression of NAFLD and NASH (**Figure 1**).

In recent years, a meta-analysis of patients with NASH demonstrated a correlation between intestinal permeability and liver damage and suggested that patients with NASH were more likely to have higher intestinal permeability and blood flow than healthy controls. The gut-liver axis, which allows intestinal bacteria and bacterial products to migrate to the liver, may be one of the underlying etiologies of NASH. In a study of 35 patients with NAFLD, Miele et al. found that NAFLD was associated with SIBO. Moreover, significant intestinal permeability was observed in 60% of the NAFLD group compared to 20.8% of the control group (*p* < 0.001). The combination of SIBO and intestinal permeability correlated with the severity of fat accumulation in the liver (31).

### 3.2 Intestinal Permeability and Gut Microbiota

The liver and gut are impacted by the nutrients and microbiome in the biliary tract, portal vein, and systemic mediators. Liver damage caused by disruption of the gut microbiome, its derived metabolites, and the gut immune system is implicated in the pathogenesis of obesity-induced IR and NAFLD. The liver is exposed to the byproducts in the portal system, which include PAMPs and damage-associated molecular patterns (DAMPs). The liver is strongly influenced by diet-induced dysbiosis. PAMPs and DAMPs induce an inflammatory response in hepatocytes, Kupffer cells, and hepatic stellate cells through a TLR cascade, which enhances the release of cytokines and chemokines, such as tumor necrosis factor (TNF)-α, IL-1, IL-6, IL-8, and interferon (IFN)-γ, and results in liver damage. Mice that were fed high-fat or choline-deficient diets demonstrated increased intestinal permeability similar to patients with NAFLD (26, 31, 32). Increased intestinal permeability triggered a proinflammatory cascade that worsened hepatic inflammation by facilitating the portal influx of microbiome-derived metabolites to the liver (32, 33). Intestinal permeability is regulated by epithelial tight junctions, which consist of several integral membrane proteins, such as zonula occludens (ZO), occludin, junctional adhesion molecule-A (JAM-A), and claudins (34). Mice that were fed HFDs exhibited decreased tight junction protein levels and increased low-grade gut inflammation as a result of microbiome abnormalities. These suggested that intestinal and gut vascular barriers were impaired by HFD-induced microbiome changes, which promoted the influx of bacterial products through the portal vein and worsened non-hepatic inflammation and metabolic abnormalities. Moreover, the mice that were fed HFDs demonstrated altered microbiota that could cross intestinal epithelial barriers, which resulted in the disruption of the intestinal epithelial and vascular barriers (31). It was unclear whether the ability to cross the damaged intestinal epithelium was an active mechanism or the result of increased intestinal permeability secondary to decreased tight junction protein expression. Several clinical studies have suggested a link between the gut microbiota, such as in SIBO and microbial dysbiosis, and the pathogenesis of NAFLD, but causality has not been established (35). Shotgun metagenomic sequencing indicated that there may be an association between *Escherichia coli* and *Bacteroides vulgatus-rich* microbiome signatures and advanced fibrosis among patients with NAFLD (36). *Escherichia* spp. were more abundant in obese children with NASH than in obese children without NASH (37). Patients with NASH (stage ≥2 fibrosis) also had significantly more *Bacteroides* and *Ruminococcus* colonies and less *Prevotella colonies* than patients without NASH, as demonstrated by 16S amplicon sequencing (38). This finding was consistent with previous evidence that indicated that *Bacteroides* and *Prevotella* are competitive species in the gut microbiota, depending on dietary composition (39).

## 4 SIBO

SIBO is a disease entity characterized by increased bacterial levels in the small intestine in amounts more typical of the large intestine. There are a number of physiologic mechanisms that prevent aberrant bacterial colonization of the small intestine, such as the acidic pH of the stomach, pancreatic enzymes, intestinal immune system, small intestine peristalsis, ileocecal valve, and intact intestinal barriers from proper construction and renewal of the intestinal walls. SIBO may develop when any one of these mechanisms are disturbed. SIBO most commonly presents with abdominal pain, diarrhea, flatulence, and abdominal overflow; however, the symptoms are usually non-specific, diverse, and may depend on the patient or etiology of SIBO. Some patients may have none of the typical intestinal symptoms but complain of weight loss, neuropathy, megaloblastic anemia, peripheral edema, erythema nodosum, and osteomalacia. SIBO has a wide variety of symptoms and often overlaps with other disease entities. As such, precise data on the prevalence of SIBO are not available. The disease is more common in the elderly and may be associated with polypharmacy, particularly with proton pump inhibitors, co-morbidities, particularly diabetes, peristaltic disorders, and endocrine disorders, and decreased gastric acid secretion.

The overgrowth of altered gut microbiota in the small intestine can affect the absorption and metabolism of carbohydrates, proteins, fats, and vitamins. Damage to the intestinal villi, impaired digestive enzyme production, and intestinal barrier dysfunction lead to malabsorption and increased nutrient loss. Larger amounts of undigested nutrients, such as sorbitol or lactose, enhance bacterial fermentation in the gut. Bile salt deconjugation is responsible for the impaired digestion and absorption of fats and fat-soluble vitamins, respectively. Anaerobic bacteria increase vitamin B12 consumption, which may result in megaloblastic anemia. Folic acid levels may increase slightly because these can be synthesized by intestinal bacteria (40, 41).

SIBO is currently diagnosed with a hydrogen breath test using glucose or lactulose (42–45). The test can be prepared, performed, and interpreted in several ways, and the results may be heterogenous across centers and physicians. Because there is a lack of a clear consensus on how this test should be performed, the results should be treated with caution. Prior to the test, antibiotics and promotility drugs and laxatives are avoided for four and one week, respectively. It is unclear whether probiotics should be avoided before testing, but proton pump inhibitors may be administered based on the North American consensus (42–45). Quantitative assessments of small intestine aspirates are not performed because the process is expensive, difficult to perform, and invasive (42, 45).

Antibiotic therapy is the primary treatment for SIBO, and rifaximin (400 mg, taken 3-4× daily for 14 days) is the first drug-of-choice. Dietary treatment and the removal of the risk factors for SIBO are also recommended (42). This treatment approach is based on the correlation between SIBO and NAFLD (46–48), and the observation that endotoxins trigger liver inflammation in mice with steatosis (49, 50). In this population of patients, the prevalence of SIBO was approximately three times that of controls. These findings correlated with those of previous studies (51). The association among SIBO, NAFLD, and endotoxemia highlight the role of the gut microbiota in the initiation and development of metabolic liver disease (26, 51).

## 5 Gut Microbiota and NAFLD

Fukui summarized gut-microbiota and NAFLD progression based on the evidence (52). Shen et al. (53) reported that Chinese NAFLD patients with moderate fibrosis (F≥2) had a higher abundance of genus Escherichia, Shigella and the corresponding Enterobacteriaceae family than those with F0/F1 mild fibrosis. Özkul et al. (54) found increased Enterobacteriaceae and decreased *Akkermansia muciniphila* (*A. muciniphila*) in their Turkish NASH patients and reported that those with moderate F ≥ 2 fibrosis also had a higher abundance of Enterobacteriaceae than those with F0/F1 fibrosis. A low abundant mucous bacteria *A. muciniphila* is known to elevate the intestinal endocannabinoids levels and to control inflammation, increase gut barrier and peptide secretion (55). This microbiome is also known to reverse high-fat diet-induced metabolic disorders, such as fat-mass gain, endotoxemia, adipose tissue inflammation, and insulin resistance (55). Boursier et al. (56) further reported that their NAFLD patients with significant fibrosis (F ≥ 2) had a higher abundance of fecal Bacteroides than those with mild F0/F1 fibrosis in France. Their NASH patients showed greater abundance of Bacteroides and Ruminococcus and smaller amount of Prevotella compared with non-NASH patients (56). Increased Bacteroides and decreased Prevotella in the feces of NASH patients is in line with above- mentioned information on the relationship between diet and gut microbiome (56) and may be regarded as the proinflammatory gut dysbiosis with the progression of NAFLD. The authors explained the effect of increased Ruminococcus by a possible increase in deleterious proinflammatory species (e.g., R. gnavus) within the genus, on the bases that the Ruminococcus genus is quite heterogeneous (56). In fact, reclassification of some proinflammatory species originally classified to Ruminococcus has been discussed (57). Furthermore, Loomba et al. (58) characterized the gut microbiome compositions using whole genome shotgun sequencing of DNA extracted from stool samples of 86 patients with biopsy-proven NAFLD and reported that Firmicutes is higher in mild/moderate NAFLD (stage 0–2 fibrosis) while Proteobacteria was higher in advanced fibrosis (stage 3 or 4 fibrosis). At the species level, the abundances of Ruminococcus obeum and Eubacterium rectale were significantly lower in advanced cases. They also found a trend of increase in *E. coli* in advanced fibrosis and demonstrated that the dysbiosis including *E. coli* dominance occurs earlier in the stage of fibrosis and may precede development of portal hypertension (58).

## 6 Endotoxin Hyperresponsiveness in NAFLD

Previous studies have shown that gut microbiota-derived endotoxins may be involved in the progression of NASH from simple fat deposition to steatohepatitis (5, 24–26, 51, 59–62). Despite these findings, the impact of increased endotoxemia on NASH progression is controversial. It is still unclear whether patients with NASH have significantly higher serum endotoxin levels than healthy controls and patients with simple fat deposition. Harte et al. reported that serum endotoxin levels were elevated in patients with NAFLD compared to healthy controls (5). In another study, patients with biopsy-proven NASH demonstrated elevated levels of plasma IgG against endotoxins, and serum IgG levels increased proportionately with NASH severity (63). These findings suggested a relationship between chronic endotoxin exposure and NASH severity in humans. Increased permeability drives endotoxemia, which triggers an inflammatory cytokine response and IR (28). In contrast, Loguercio et al. (5, 64) did not identify endotoxemia in any patient with NAFLD in their study; however, the results of their study were inconsistent. Currently, there is a general agreement that mild portal endotoxemia from gut-derived bacterial endotoxins can be detected in healthy participants (26); however, mild portal endotoxemia does not usually cause liver dysfunction (65). We propose that low-level endotoxin-mediated mechanisms may contribute to the progression of NASH. Here, we hypothesized that patients with simple adiposity may demonstrate enhanced responsiveness to gut-derived bacterial endotoxins compared to healthy controls. Furthermore, our data showed that HFD-induced murine adiposity enhanced the response to low-dose LPS. Low-dose LPS resulted in liver injury and severe liver fibrosis in HFD-fed mice but not chow-fed mice (66). Previous studies have also shown that a high-cholesterol diet increases the sensitivity of mice to LPS even in the absence of changes to plasma levels of LPS. This further supported our hypothesis (67). Cluster of differentiation (CD)14 enhances the effect of LPSs in Kupffer cells and is an important regulatory factor in LPS-induced inflammation (68–73). A previous report suggested that the promoter polymorphisms of CD14 were risk factors for human NASH (74). As such, the increased expression of CD14 is closely related to the pathogenesis of NASH. Indeed, our data demonstrated that patients with NAFLD, NAFL, and NASH expressed more CD14 mRNA than healthy controls (66). Hepatic CD14 may serve as an important factor in the development of NASH by enhancing hepatic inflammation against gut-derived bacterial endotoxins. We also investigated the leptin-dependent increase in hepatic CD14 expression in leptin-deficient ob/ob mice and leptin receptor-deficient db/db mice. Leptin and signal transducer and activation of transcription (STAT)3 signaling increased the number of CD14-positive Kupffer cells, which increased the responsiveness to gut-derived, low-dose bacterial endotoxins, regardless of the presence of steatosis. In humans, elevated serum leptin levels are associated with obesity, visceral fat accumulation, and fat deposition (75, 76). Enhanced expression of leptin-induced hepatic CD14 may increase hepatic responsiveness to even low levels of gut microbiota-derived endotoxins, which may prompt the progression of simple steatosis to NASH *via* STAT3 signaling. Moreover, our previous study demonstrated that resveratrol, a natural polyphenol, reduced inflammation and fibrosis by controlling CD14 expression in Kupffer cells, which inhibited LPS reactivity. These findings suggested that resveratrol may be a treatment option for NASH (77).

## 7 Therapeutic Approach for Reducing LPS in NAFLD

Few studies have assessed the relationship between dietary interventions and serum LPS levels. Healthy dietary patterns seem to be associated with lower serum LPS activity. The Mediterranean diet, which is rich in fiber and unsaturated fats, has been recommended to reduce endotoxemia (78). The Finn Dianne Study conducted a nutritional survey of 668 individuals with type 1 diabetes and found that healthy dietary choices, such as the consumption of fish, fresh vegetables, and fruits and berries, were associated with reduced systemic endotoxemia (79). This study also placed eight healthy participants on a Western-style diet for one month. This subgroup of patients demonstrated a 71% increase in plasma endotoxin activity, whereas patients on a prudent diet reduced endotoxin activity levels by 31% (6). Another study examined the effect of diet on LPS fasting plasma levels in 20 older adults. This study demonstrated that a low-fat, high-carbohydrate diet enriched in n-3 polyunsaturated fatty acids reduced LPS fasting plasma levels compared to Mediterranean and high-saturated fat diets (0.24 ± 0.01 EU/mL *vs.* 0.38 ± 0.06 EU/mL and 0.35 ± 0.03 EU/mL, respectively) (80).

Postprandial endotoxemia occurs after meals and is characterized by different fatty acid compositions. Several studies on postprandial endotoxemia have shown that high-saturated fat meals increase serum LPS levels, which suggested that dietary fats have different regulatory effects on intestinal epithelial endotoxin transport (81) and postprandial low-grade inflammation (82, 83). While evidence supports a positive association between HFDs and endotoxemia, the link among dietary patterns, intestinal microbiota, subclinical inflammation, and endotoxemia is still debated (84).

Probiotics or prebiotics regulate the gut flora and are newly recommended for the prevention and treatment of several metabolic diseases, such as NAFLD. Probiotic bacteria reduce pathogenic bacterial growth and restore the integrity of the intestinal barrier against LPS-induced epithelial toxicity (85).

Previous data demonstrated that serum LPS, liver TLR4-mRNA, and serum inflammatory cytokines were all significantly decreased in the probiotic intervention group compared to the NAFLD model group. Additionally, the intervention group demonstrated less liver steatosis and inflammatory cell infiltration compared to the model group. These results supported the hypothesis that probiotics may delay the process of NAFLD by inhibiting the LPS–TLR4 signaling pathway (85). The narrative review by Eslamparast et al. summarized studies that demonstrated that probiotic supplementation improved the inflammatory status and clinical manifestations of NAFLD in animal and human models (86). Recently, the dose-dependent effects of multispecies probiotic supplementation on serum LPS levels and cardiometabolic profiles in obese postmenopausal women have been demonstrated (87).

Many antibiotics have regulatory effects on the intestinal microbiota and are beneficial to NAFLD (88). For example, oral cidomycin increased small intestine transit rates and reduced serum levels of alanine aminotransferase (ALT), aspartate aminotransferase (AST), and TNF-α in a NASH mouse model, which suggested that cidomycin may reduce the severity of NASH by modulating intestinal microbiota (59). Rifaximin, a largely water-insoluble and nonabsorbable (<0.4%) drug, has been shown to exert antimicrobial activity against enteric bacteria such as *Streptococcus*, *Bacteroides*, and *Citrobacter* (89). Gangarapu et al. demonstrated that short-term administration of rifaximin (1,200 mg/day for 28 days) improved the clinical status of patients with NAFLD/NASH and reduced serum transaminase and circulating endotoxin levels (90). Abdel-Razik et al. reported that patients with NASH demonstrated significantly reduced proinflammatory cytokine and ALT levels and lower NAFLD-liver fat scores after rifaximin therapy (1,100 mg/day for six months) (91). In contrast, an open-label clinical trial suggested that rifaximin administration (800 mg/day for six weeks) was not effective in humans with NASH (92). The inconsistency may be because of the small sample size, relatively low treatment dose, or short duration of this clinical study.

## 8 Treatment for SIBO and Gut-Barrier in NAFLD

**Figure 2:**
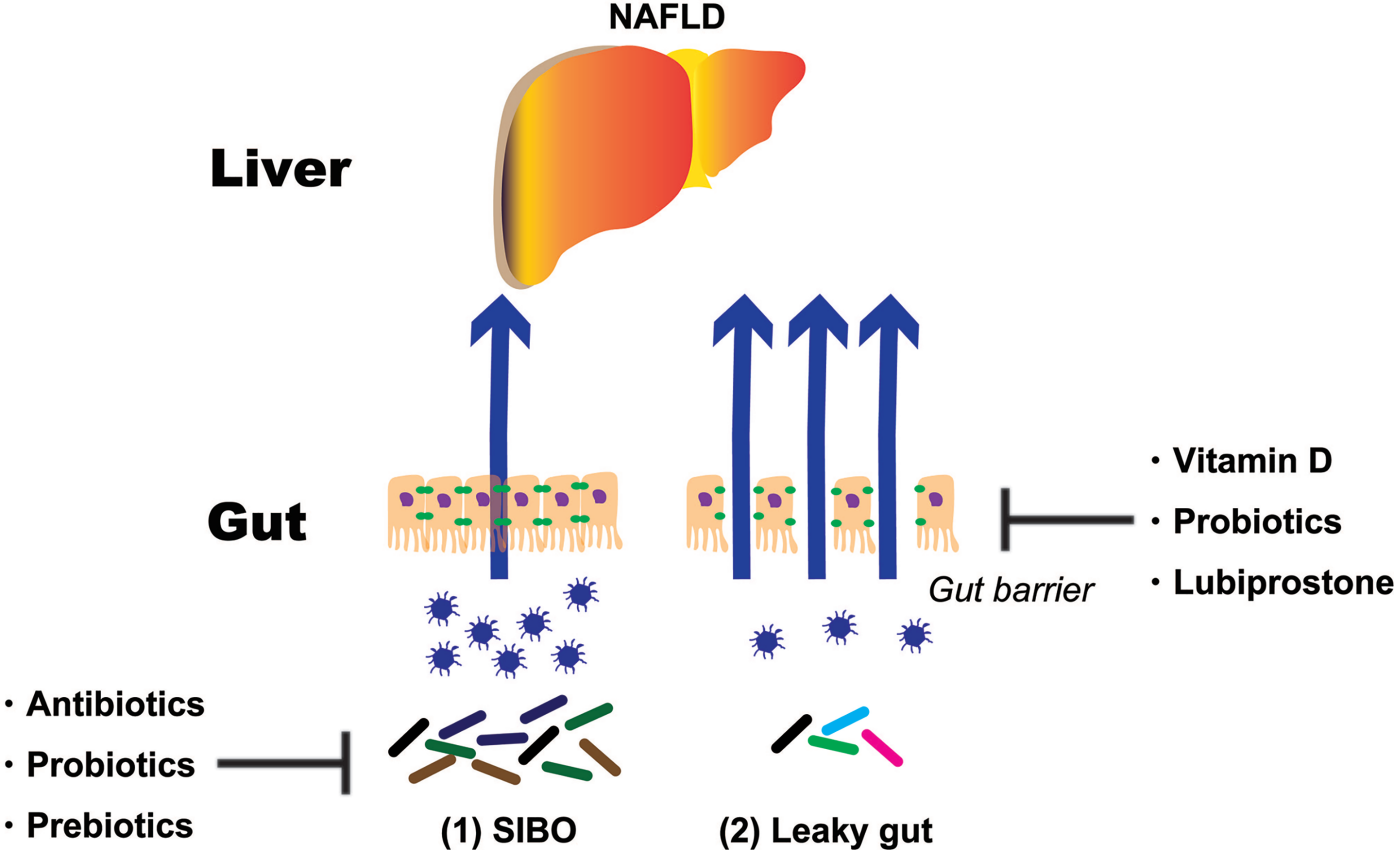
Treatment strategies targeting the intestinal tract of NAFLD patients. NAFLD, non-alcoholic fatty liver disease; SIBO, small intestinal bacterial overgrowth.

As mentioned above, NAFLD treatments targeting the intestinal tract can be broadly divided into two categories. One is SIBO and the second is an intestinal barrier. This paper reports evidence for SIBO and intestinal barriers (**Figure 2**).

### 8.1 Treatment of SIBO and Its Impact on the Course of NAFLD

#### 8.1.1 Antibiotics

Only antibiotics that work in the gastrointestinal tract alone, such as rifaximin (1,200–1,600 mg/day for 14 days) and metronidazole (750 mg/day for 10–14 days), are prescribed for SIBO. Antibiotic therapy can be repeated for disease relapse. Rifaximin has broad-spectrum activity against gram-positive and gram-negative aerobic and anaerobic bacteria. It exhibits bile acid-dependent solubility and is more effective in the small intestine than the colon (93). In particular, rifaximin seems to exert stable and persistent changes to the composition of duodenal bacteria, whereas its effects on colonic gut microbiota are gradually and completely reversed following treatment interruption (94). Rifaximin has bactericidal and bacteriostatic activity, which is typical of antibiotics, but rifaximin also exerts non-traditional effects by positively modulating the composition and interactions of the microbiota (42, 92). Rifaximin downregulates the inflammatory and NF-κB responses by inhibiting proinflammatory cytokine and pregnane X receptor (PXR) activation, respectively (95, 96). Rifaximin has been proven to be beneficial for severe liver disease and the secondary prevention of hepatic encephalopathy. It also contributes to the secondary prevention of spontaneous bacterial peritonitis and works as an adjuvant to beta-blockers to reduce portal vein system pressure, which significantly reduces the risk of bleeding from esophageal varices (41). The drug is commonly prescribed in clinical practice; however, there is less available data on its efficacy in NAFLD. Gangarapu et al. prescribed rifaximin (1,200 mg/day for 28 days) to 42 patients with biopsy-confirmed NAFLD, which included 27 patients with NASH. After the administration of rifaximin, there was a significant decrease in the AST, γ-glutamyl transpeptidase, and endotoxemia levels of the patients with NASH (90).

#### 8.1.2 Probiotics

Experiments were conducted on animals on VSL#3 (Alfasigma USA, Inc., Covington, La, USA), which is comprised of Streptococcus thermophilus, Bifidobacterium longum, Bifidobacterium breve, Bifidobacterium infantis, Lactobacillus acidophilus, Lactobacillus plantarum, Lactobacillus paracasei, and Lactobacillus delbrueckii ssp. Bulgaricus. VSL#3 (Alfasigma USA, Inc.) has shown promising results for animal models of NAFLD by decreasing the severity of histopathological changes and activity of aminotransferases (97); however, its benefits in human models are equivocal. Loguercio et al. administered VSL#3 (Alfasigma USA, Inc.) to 22 and 20 patients with NAFLD and alcoholic cirrhosis, respectively, and demonstrated reductions in serum oxidative stress factors (98). Malaguarmera et al. categorized 66 patients with NASH into two subgroups. Both groups were advised lifestyle modifications, but one group was additionally treated with Bifidobacterium longum and fructooligosaccharides. The group treated with the prebiotics demonstrated significant decreases in TNF-α, C-reactive protein, low-density lipoprotein cholesterol, and endotoxin concentrations. Patients were evaluated based on their AST and NALFD activity, homeostatic model assessment for insulin resistance score, and steatosis grade (99). Wong et al. administered Lactobacillus and Bifidobacterium spp. to 16 patients with NASH. The patients demonstrated reductions in steatosis levels after treatment but no changes in the other morphologic parameters (100). As previously reported, quantitative assessments of the individual bacteria in patients with NAFLD/NASH are inconsistent. The administered probiotics did not contain bacterial strains, the greatest deficiency of which is observed in these diseases.

Prebiotics are non-digestible nutrients improved the composition of the gut microbiota and regenerating the intestinal epithelium. Probiotics primarily treat NAFLD by improving lipid metabolism. Probiotics also change the composition of the microbiota, improve the intestinal barrier, reduce intestinal permeability, and lower the levels of proinflammatory cytokines, which reduce bacterial translocation. There are inadequate studies on the efficacy of probiotics, but probiotics may be considered in patients who do not respond to conventional therapy. A low-calorie diet with reduced carbohydrate and fat and increased choline content may have a beneficial effect on the composition of the intestinal microbiota, but no current studies can support this hypothesis (97). It is highly probable that such a diet retards the development of NAFLD and should be recommended for patients with this disease.

### 8.2 Therapeutic Target of Gut Barrier for NAFLD

#### 8.2.1 Vitamin D

NAFLD also seems to be caused by vitamin D deficiency, but the underlying mechanisms are poorly understood. Vitamin D has recently been identified as a possible factor in the dysregulation of the gut-liver axis. Optimal vitamin D levels maintain the intestinal barrier by up-regulating the production of tight junction components and mucosal proteoglycans in the ileal epithelium. Vitamin D also reduces microbial proliferation by promoting intestinal mucosal Paneth cells to produce defensins and converting enzymes, such as matrix metalloproteinase 7. Vitamin D deficiency in an HFD mouse model exacerbated a leaky gut, enterotoxemia, endotoxemia, and systemic inflammation, which worsened IR and hepatic lipidosis (101). Thus, supplementation with vitamin D has been recommended (102).

#### 8.2.2 Prebiotics

Prebiotics may have beneficial effects on NAFLD and NASH. In animal models, prebiotics altered the gut microbiota composition and increased plasma glucagon-like peptide(GLP)-2 levels, which improved gut barrier function. Prebiotics also reduce liver inflammation and treat the metabolic disorders associated with obesity and diabetes (103). Prebiotics, such as inulin and oligofructose, control the growth of *Faecalibacterium prausnitzii* and *Bifidobacterium* and reduce plasma endotoxin levels by increasing GLP-1 secretion and the trophic effect of GLP-2 on gut barrier integrity (104). In humans, oligofructose supplementation improves glucose tolerance and promotes weight loss by regulating the expression of hormones involved in energy intake, such as ghrelin and peptide YY, in obesity (105). A meta-analysis of probiotic, prebiotic, and synbiotic therapies for NAFLD demonstrated that these treatments significantly reduced BMI, ALT, and AST levels. Compared to prebiotics and probiotics, synbiotics did not decrease serum lipid levels (106).

Biogenic heat-killed lactic acid bacteria have been used in studies of NAFLD and NASH, because they are easier to handle than live lactic acid bacteria. Heat-killed *Lactobacillus reuteri* GMNL-263 (Lr263; Genmont Biotech Incorporation, Tainan, Taiwan) reduced cardiac and liver fibrosis in HFD-fed mice by suppressing tumor growth factor (TGF)-β (107). Live Lr263 (Genmont Biotech Incorporation) also improved inflammation, IR, and hepatic steatosis in high fructose-fed rats (108). In comparison, heat-killed *Lactobacillus plantarum* L-137 (HK L-137; House Wellness Foods Corp., Hyogo, Japan), which was isolated from fermented fish and rice, attenuated adipose tissue and hepatic inflammation in DahlS. *Z-Leprfa/Leprfa* rats are the model for metabolic syndrome (109). The live and heat-killed *Lactobacillus pentosus* strains, which are isolated from Kyoto pickles called *shibazuke*, reportedly enhanced splenic natural killer (NK) activity and IFN-γ production in mice (110, 111). Heat-killed S-PT84 partially restored the expression of ZO-1, occludin, and xlaudin-3 but did not restore alterations in the microbiota profile of a NASH model. Heat-killed S-PT84 suppressed metabolic endotoxemia by maintaining the gut barrier, regulating intestinal permeability, and suppressing IL-17-producing T(Th17)-cell accumulation in the intestinal lamina propria. While heat-killed S-PT84 had no effect on the abundance of NK T-cells in the liver, it attenuated hepatic inflammation and fibrosis by decreasing the macrophage (M)1/M2 ratio in the liver. These results indicated that heat-killed S-PT84 attenuated lipotoxicity-induced hepatic IR and steatohepatitis in a NASH animal model (112). In contrast, live *Lactobacillus pentosaceus* LP28 isolated from the longan fruit (*Euphoria longana*) reduced weight gain and liver triglyceride and cholesterol levels in HFD-fed mice. Heat-killed LP28 did not prevent metabolic syndrome (113). Similar to other live lactic acid bacteria, biogenic heat-killed lactic acid bacteria can improve NAFLD and NASH. The immunomodulatory effects, shelf-life, and storage and transportation of heat-killed lactic acid bacteria should be further investigated in clinical and animal studies.

#### 8.2.3 Constipation Drug Lubiprostone

**Figure 3:**
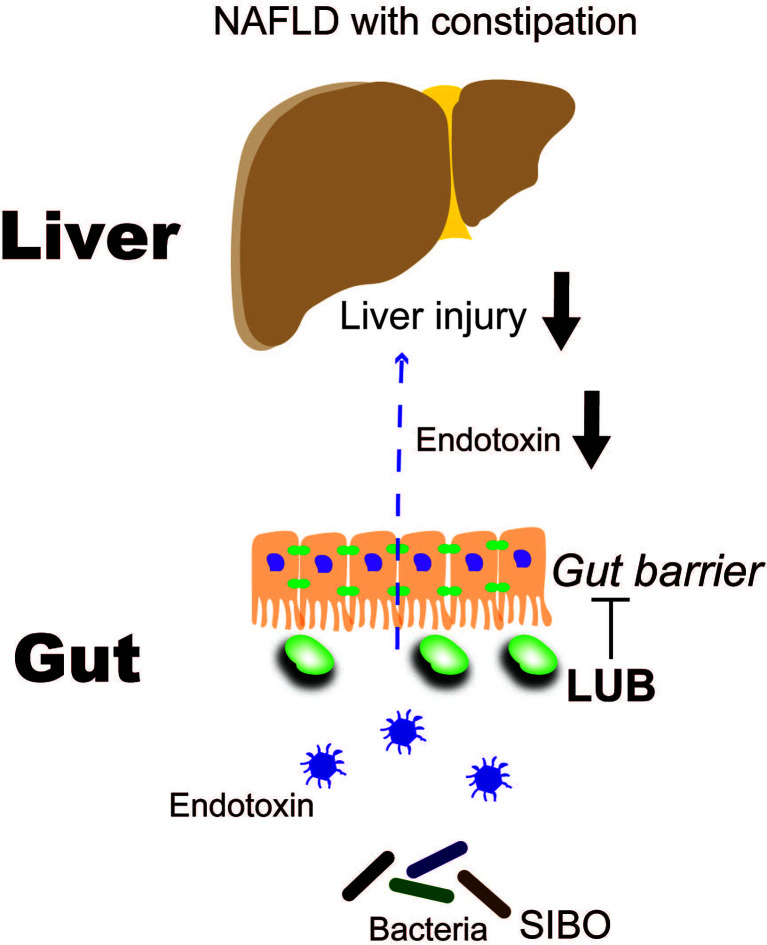
Lubiprostone exerts therapeutic effects on NAFLD by targeting the intestinal barrier (19). LUB, lubiprostone; NAFLD, non-alcoholic fatty liver disease; SIBO, small intestinal bacterial overgrowth.

LUB is a bicyclic fatty acid derived from the prostone metabolite, prostaglandin E1. It is a type 2 chloride channel activator that promotes chloride efflux into the gastrointestinal lumen, which results in intestinal fluid secretion (114). LUB is typically prescribed to treat chronic idiopathic constipation and irritable bowel syndrome with constipation, but it has additional potential actions on the intestinal mucosa. For example, LUB prevented non-steroidal anti-inflammatory drug (NSAID)-induced small bowel injury in rats (115), ameliorated increased intestinal permeability in an atherosclerotic mouse model that was fed a Western diet (116), and maintained intestinal tight junction barrier functions by activating the chloride channel in the Caco-2 cell line (117). We previously reported that LUB dramatically reduced NSAID-induced intestinal permeability in healthy controls (16). A recent three-arm parallel, double-blind, randomized controlled trial examined LUB as a new therapeutic target for NAFLD. A total of 150 Japanese patients with NAFLD and constipation were treated with a placebo or 12 μg or 24 μg of LUB for 12 weeks. The LUB groups demonstrated improvements in the urinary lactulose/mannitol ratio, which is an index of intestinal permeability. Liver enzymes, liver fat, and blood endotoxin levels were significantly improved (19). In particular, the patients with improved intestinal permeability demonstrated a marked decrease in liver enzymes levels, liver adipose content, and blood endotoxin concentration (**Figure 3**). Drugs that target intestinal permeability may be a promising new therapeutic approach for NAFLD.

## 9 Conclusion and Outlook

The pathogenesis and progression of NAFLD/NASH are associated with increased susceptibility to endotoxins, increased intestinal permeability, and qualitative and quantitative abnormalities in intestinal bacteria. Advances in the understanding of the role of the gut-liver axis in NAFLD pathogenesis and the encouraging results of gut microbiota modulation with probiotic supplementation provide promising and safe alternatives for therapy. Additional extensive and long-term studies are needed to better identify the best probiotic strains and the dose, timing, and duration of supplementation therapy. These will allow physicians to individualize probiotic therapy using a patient-tailored approach to modulate intestinal permeability and endotoxemia and treat liver disease. Future studies should explore the pathogenesis of NAFLD, particularly the mechanisms behind intestinal permeability, to facilitate the development of novel therapeutic approaches that target the gut-liver axis.

## Author Contributions

Conceptualization, TKe and Ana. Writing—original draft preparation, TKe, TKo, and MI. Writing—review and editing, KI, KTan, AY, KTak, YK, AO, MI, ANo, YH, YO, SK, TH, KH, MY, NK, and SS. Supervision, TO, HU, and KW. All authors have read and agreed to the published version of the manuscript.

## Conflict of Interest

ANa receives grants and research support from Gilead, Mylan EPD, EA Pharma, Kowa, Taisho, and Biofermin. ANa is also a consultant for Gilead, Boehringer Ingelheim, Bristol Myers Squibb, Kowa, Astellas, EA Pharma, and Mylan EPD.

The remaining authors declare that the research was conducted in the absence of any commercial or financial relationships that could be construed as a potential conflict of interest.

## Publisher’s Note

All claims expressed in this article are solely those of the authors and do not necessarily represent those of their affiliated organizations, or those of the publisher, the editors and the reviewers. Any product that may be evaluated in this article, or claim that may be made by its manufacturer, is not guaranteed or endorsed by the publisher.

## References

[1] Loomba R Sanyal AJ. The Global NAFLD Epidemic. Nat Rev Gastroenterol Hepatol (2013) 10:686–90. doi: 10.1038/nrgastro.2013.171 24042449

[2] Younossi ZM Koenig AB Abdelatif D Fazel Y Henry L Wymer M. Global Epidemiology of Nonalcoholic Fatty Liver Disease-Meta-Analytic Assessment of Prevalence, Incidence, and Outcomes. Hepatology (2016) 64:73–84. doi: 10.1002/hep.28431 26707365

[3] Clemente MG Mandato C Poeta M Vajro P. Pediatric Non-Alcoholic Fatty Liver Disease: Recent Solutions, Unresolved Issues, and Future Research Directions. World J Gastroenterol (2016) 22:8078–93. doi: 10.3748/wjg.v22.i36.8078 PMC5037077 27688650

[4] Rotman Y Sanyal AJ. Current and Upcoming Pharmacotherapy for Non-Alcoholic Fatty Liver Disease. Gut (2017) 66:180–90. doi: 10.1136/gutjnl-2016-312431 27646933

[5] Harte AL da Silva NF Creely SJ McGee KC Billyard T Youssef-Elabd EM. Elevated Endotoxin Levels in Non-Alcoholic Fatty Liver Disease. J Inflammation (Lond) (2010) 7:15. doi: 10.1186/1476-9255-7-15 PMC2873499 20353583

[6] Pendyala S Walker JM Holt PR. A High-Fat Diet Is Associated With Endotoxemia That Originates From the Gut. Gastroenterology (2012) 142:1100–1.e2. doi: 10.1053/j.gastro.2012.01.034 22326433 PMC3978718

[7] O’Neill LA Bowie AG. The Family of Five: TIR-Domaincontaining Adaptors in Toll-Like Receptor Signalling. Nat Rev Immunol (2007) 7:353–64. doi: 10.1038/nri2079 17457343

[8] Kagan JC Medzhitov R. Phosphoinositide-Mediated Adaptor Recruitment Controls Toll-Like Receptor Signaling. Cell (2006) 125:943–55. doi: 10.1016/j.cell.2006.03.047 16751103

[9] Fitzgerald KA Chen ZJ. Sorting Out Toll Signals. Cell (2006) 125:834–36. doi: 10.1016/j.cell.2006.05.014 16751092

[10] Seki E Brenner DA. Toll-Like Receptors and Adaptor Molecules in Liver Disease: Update. Hepatology (2008) 48:322–35. doi: 10.1002/hep.22306 18506843

[11] Bianchi ME. DAMPs, PAMPs and Alarmins: All We Need to Know About Danger. J Leukoc Biol (2007) 81:1–5. doi: 10.1189/jlb.0306164 17032697

[12] Szabo G Dolganiuc A Mandrekar P. Pattern Recognition Receptors: A Contemporary View on Liver Diseases. Hepatology (2006) 44:287–98. doi: 10.1002/hep.21308 16871558

[13] Kuula H Salo T Pirilä E Tuomainen AM Jauhiainen M Uitto V-J. Local and Systemic Responses in Matrix Metalloproteinase 8-Deficient Mice During Porphyromonas Gingivalis-Induced Periodontitis. Infect Immun (2009) 77:850–9. doi: 10.1128/IAI.00873-08 PMC2632031 19029300

[14] Kalambokis GN Mouzaki A Rodi M Pappas K Korantzopoulos P Tsianos E. Circulating Endotoxin and Interleukin-6 Levels Are Associated With Doppler-Evaluated Pulmonary Vascular Resistance in Cirrhotic Patients. Hepatol Int (2012) 6:783–9. doi: 10.1007/s12072-011-9337-0 26201526

[15] Tsukamoto H Fukudome K Takao S Tsuneyoshi N Kimoto M. Lipopolysaccharide-Binding Protein-Mediated Toll-Like Receptor 4 Dimerization Enables Rapid Signal Transduction Against Lipopolysaccharide Stimulation on Membrane-Associated CD14-Expressing Cells. Int Immunol (2010) 22:271–80. doi: 10.1093/intimm/dxq005 20133493

[16] Kato T Honda Y Kurita Y Iwasaki A Sato T Kessoku T. Lubiprostone Improves Intestinal Permeability in Humans, a Novel Therapy for the Leaky Gut: A Prospective Randomized Pilot Study in Healthy Volunteers. PloS One (2017) 12:e0175626. doi: 10.1371/journal.pone.0175626 28410406 PMC5391961

[17] Marshall JC Foster D Vincent JL Cook DJ Cohen J Dellinger RP. Diagnostic and Prognostic Implications of Endotoxemia in Critical Illness: Results of the MEDIC Study. J Infect Dis (2004) 190:527–34. doi: 10.1086/422254 15243928

[18] Romaschin AD Harris DM Ribeiro MB Paice J Foster DM Walker PM. A Rapid Assay of Endotoxin in Whole Blood Using Autologous Neutrophil Dependent Chemiluminescence. J Immunol Methods (1998) 212:169–85. doi: 10.1016/S0022-1759(98)00003-9 9672205

[19] Kessoku T Imajo K Kobayashi T Ozaki A Iwaki M Honda Y. Lubiprostone in Patients With Non-Alcoholic Fatty Liver Disease: A Randomised, Double-Blind, Placebo-Controlled, Phase 2a Trial. Lancet Gastroenterol Hepatol (2020) 5:996–1007. doi: 10.1016/S2468-1253(20)30216-8 32805205

[20] Bjarnason I Takeuchi K Bjarnason A Adler SN Teahon K. The G.U.T. of Gut. Scand J Gastroenterol (2004) 39:807–15. doi: 10.1080/00365520410003326 15513377

[21] Zeuzem S. Gut-Liver Axis. Int J Colorectal Dis (2000) 15:59–82. doi: 10.1007/s003840050236 10855547

[22] Dumas ME Barton RH Toye A Cloarec O Blancher C Rothwell A. Metabolic Profiling Reveals a Contribution of Gut Microbiota to Fatty Liver Phenotype in Insulin-Resistant Mice. Proc Natl Acad Sci USA (2006) 103:12511–6. doi: 10.1073/pnas.0601056103 PMC1567909 16895997

[23] Solga SF Diehl AM. Non-Alcoholic Fatty Liver Disease: Lumen-Liver Interactions and Possible Role for Probiotics. J Hepatol (2003) 38:681–7. doi: 10.1016/S0168-8278(03)00097-7 12713883

[24] Farhadi A Gundlapalli S Shaikh M Frantzides C Harrell L Kwasny MM. Susceptibility to Gut Leakiness: A Possible Mechanism for Endotoxaemia in Non-Alcoholic Steatohepatitis. Liver Int (2008) 28:1026–33. doi: 10.1111/j.1478-3231.2008.01723.x PMC4303249 18397235

[25] Brun P Castagliuolo I Di Leo V Buda A Pinzani M Palù G. Increased Intestinal Permeability in Obese Mice: New Evidence in the Pathogenesis of Nonalcoholic Steatohepatitis. Am J Physiol Gastrointest Liver Physiol (2007) 292:G518–25. doi: 10.1152/ajpgi.00024.2006 17023554

[26] Miele L Valenza V La Torre G Montalto M Cammarota G Ricci R. Increased Intestinal Permeability and Tight Junction Alterations in Nonalcoholic Fatty Liver Disease. Hepatology (2009) 49:1877–87. doi: 10.1002/hep.22848 19291785

[27] Cani PD Bibiloni R Knauf C Waget A Neyrinck AM Delzenne NM. Changes in Gut Microbiota Control Metabolic Endotoxemia-Induced Inflammation in High-Fat Diet-Induced Obesity and Diabetes in Mice. Diabetes (2008) 57:1470–81. doi: 10.2337/db07-1403 18305141

[28] Cani PD Amar J Iglesias MA Poggi M Knaug C Bastelica D. Metabolic Endotoxemia Initiates Obesity and Insulin Resistance. Diabetes (2007) 56:1761–72. doi: 10.2337/db06-1491 17456850

[29] Sharma R Young C Neu J. Molecular Modulation of Intestinal Epithelial Barrier: Contribution of Microbiota. J BioMed Biotechnol (2010) 2010:305879. doi: 10.1155/2010/305879 20150966 PMC2817557

[30] Ghoshal S Witta J Zhong J de Villiers W Eckhardt E. Chylomicrons Promote Intestinal Absorption of Lipopolysaccharides. J Lipid Res (2009) 50:90–7. doi: 10.1194/jlr.M800156-JLR200 18815435

[31] Mouries J Brescia P Silvestri A Spadoni I Sorribas M Wiest R. Microbiota-Driven Gut Vascular Barrier Disruption Is a Prerequisite for Non-Alcoholic Steatohepatitis Development. J Hepatol (2019) 71:1216–28. doi: 10.1016/j.jhep.2019.08.005 PMC6880766 31419514

[32] Albillos A de Gottardi A Rescigno M. The Gut-Liver Axis in Liver Disease: Pathophysiological Basis for Therapy. J Hepatol (2020) 72:558–77. doi: 10.1016/j.jhep.2019.10.003 31622696

[33] Tilg H Moschen AR Szabo G. Interleukin-1 and Inflammasomes in Alcoholic Liver Disease/Acute Alcoholic Hepatitis and Nonalcoholic Fatty Liver Disease/Nonalcoholic Steatohepatitis. Hepatology (2016) 64:955–65. doi: 10.1002/hep.28456 26773297

[34] Gonzalez-Mariscal L Betanzos A Nava P Jaramillo BE. Tight Junction Proteins. Prog Biophys Mol Biol (2003) 81:1–44. doi: 10.1016/S0079-6107(02)00037-8 12475568

[35] Wieland A Frank DN Harnke B Bambha K. Systematic Review: Microbial Dysbiosis and Nonalcoholic Fatty Liver Disease. Aliment Pharmacol Ther (2015) 42:1051–63. doi: 10.1111/apt.13376 26304302

[36] Loomba R Seguritan V Li W Long T Klitgord N Bhatt A. Gut Microbiome-Based Metagenomic Signature for Non-Invasive Detection of Advanced Fibrosis in Human Nonalcoholic Fatty Liver Disease. Cell Metab (2019) 30:607. doi: 10.1016/j.cmet.2019.08.002 31484056 PMC8025688

[37] Zhu L Baker SS Gill C Liu W Alkhouri R Baker RD. Characterization of Gut Microbiomes in Nonalcoholic Steatohepatitis (NASH) Patients: A Connection Between Endogenous Alcohol and NASH. Hepatology (2013) 57:601–9. doi: 10.1002/hep.26093 23055155

[38] Boursier J Mueller O Barret M Machado M Fizanne L Araujo-Perez F. The Severity of Nonalcoholic Fatty Liver Disease Is Associated With Gut Dysbiosis and Shift in the Metabolic Function of the Gut Microbiota. Hepatology (2016) 63:764–75. doi: 10.1002/hep.28356 PMC4975935 26600078

[39] Gophna U. Microbiology. The Guts of Dietary Habits. Science (2011) 334:45–6. doi: 10.1126/science.1213799 21980098

[40] Gasbarrini A Lauritano EC Gabrielli M Scarpellini E Lupascu A Ojetti V. Small Intestinal Bacterial Overgrowth: Diagnosis and Treatment. Dig Dis (2007) 25:237–40. doi: 10.1159/000103892 17827947

[41] Fan X Sellin JH. Review Article: Small Intestinal Bacterial Overgrowth, Bile Acid Malabsorption and Gluten Intolerance as Possible Causes of Chronic Watery Diarrhoea. Aliment Pharmacol Ther (2009) 29:1069–77. doi: 10.1111/j.1365-2036.2009.03970.x 19222407

[42] Boltin D Perets TT Shporn E Aizic S Levy S Niv Y. Rifaximin for Small Intestinal Bacterial Overgrowth in Patients Without Irritable Bowel Syndrome. Ann Clin Microbiol Antimicrob (2014) 13:49. doi: 10.1186/s12941-014-0049-x 25319626 PMC4201689

[43] Quigley EM. Small Intestinal Bacterial Overgrowth: What It Is and What It Is Not. Curr Opin Gastroenterol (2014) 30:141–6. doi: 10.1097/MOG.0000000000000040 24406476

[44] Rezaie A Buresi M Lembo A Lin H McCallum R Rao S. Hydrogen and Methane-Based Breath Testing in Gastrointestinal Disorders: The North American Consensus. Am J Gastroenterol (2017) 112:775–84. doi: 10.1038/ajg.2017.46 PMC5418558 28323273

[45] Usai-Satta P Giannetti C Oppia F Cabras F. The North American Consensus on Breath Testing: The Controversial Diagnostic Role of Lactulose in SIBO. Am J Gastroenterol (2018) 113:440. doi: 10.1038/ajg.2017.392 29535443

[46] Nazim M Stamp G Hodgson HJ. Non-Alcoholic Steatohepatitis Associated With Small Intestinal Diverticulosis and Bacterial Overgrowth. Hepatogastroenterology (1989) 36:349–51. 2516007

[47] Lichtman SN Sartor RB Keku J Schwab JH. Hepatic Inflammation in Rats With Experimental Small Intestinal Bacterial Overgrowth. Gastroenterology (1990) 98:414–23. doi: 10.1016/0016-5085(90)90833-M 2295397

[48] Lichtman SN Keku J Schwab JH Sartor RB. Hepatic Injury Associated With Small Bowel Bacterial Overgrowth in Rats Is Prevented by Metronidazole and Tetracycline. Gastroenterology (1991) 100:513–9. doi: 10.1016/0016-5085(91)90224-9 1985047

[49] Diehl AM Li ZP Lin HZ Yang SQ. Cytokines and the Pathogenesis of Non-Alcoholic Steatohepatitis. Gut (2005) 54:303–6. doi: 10.1136/gut.2003.024935 PMC1774847 15647199

[50] Farrell GC Larter CZ. Nonalcoholic Fatty Liver Disease: From Steatosis to Cirrhosis. Hepatology (2006) 43:S99–S112. doi: 10.1002/hep.20973 16447287

[51] Wigg AJ Roberts-Thomson IC Dymock RB McCarthy PJ Grose RH Cummins AG. The Role of Small Intestinal Bacterial Overgrowth, Intestinal Permeability, Endotoxaemia, and Tumour Necrosis Factor Alpha in the Pathogenesis of Non-Alcoholic Steatohepatitis. Gut (2001) 48:206–11. doi: 10.1136/gut.48.2.206 PMC1728215 11156641

[52] Fukui H. Role of Gut Dysbiosis in Liver Diseases: What Have We Learned So Far? Diseases (2019) 7:58. doi: 10.3390/diseases7040058 PMC6956030 31726747

[53] Shen F Zheng RD Sun XQ Ding WJ Wang XY Fan JG. Gut Microbiota Dysbiosis in Patients With Non-Alcoholic Fatty Liver Disease. Hepatobiliary Pancreatic Dis Int (2017) 16:375–81. doi: 10.1016/S1499-3872(17)60019-5 28823367

[54] Özkul C Yalinay M Karakan T Yilmaz G. Determination of Certain Bacterial Groups in Gut Microbiota and Endotoxin Levels in Patients With Nonalcoholic Steatohepatitis. Turkish J Gastroenterol (2017) 28:361–69. doi: 10.5152/tjg.2017.17033 28705785

[55] Everard A Belzer C Geurts L Ouwerkerk JP Druart C Bindels LB. Cross-Talk Between Akkermansia Muciniphila and Intestinal Epithelium Controls Diet-Induced Obesity. Proc Natl Acad Sci USA (2013) 110:9066–71. doi: 10.1073/pnas.1219451110 PMC3670398 23671105

[56] Boursier J Mueller O Barret M Machado M Fizanne L Araujo-Perez F. The Severity of Nonalcoholic Fatty Liver Disease Is Associated With Gut Dysbiosis and Shift in the Metabolic Function of the Gut Microbiota. Hepatology (2016) 63:764–75. doi: 10.1002/hep.28356 PMC4975935 26600078

[57] Hammes TO Leke R Escobar TDC Fracasso LB Meyer FS Andrades MÉ.. Lactobacillus rhamnosusGG Reduces Hepatic Fibrosis in a Model of Chronic Liver Disease in Rats. Nutricion Hospitalaria (2017) 34:702–9. doi: 10.20960/nh.626 28627210

[58] Loomba R Seguritan V Li W Long T Klitgord N Bhatt A. Gut Microbiome-Based Metagenomic Signature for Non-Invasive Detection of Advanced Fibrosis in Human Nonalcoholic Fatty Liver Disease. Cell Metab (2017) 25:1054–62. doi: 10.1016/j.cmet.2017.04.001 PMC5502730 28467925

[59] Wu WC Zhao W Li S. Small Intestinal Bacteria Overgrowth Decreases Small Intestinal Motility in the NASH Rats. World J Gastroenterol (2008) 14:313–7. doi: 10.3748/wjg.14.313 PMC2675133 18186574

[60] Zhao LF Jia JM Han DW. The Role of Enterogenous Endotoxemia in the Pathogenesis of Non-Alcoholic Steatohepatitis. Zhonghua Gan Zang Bing Za Zhi (2004) 12:632. 15504303

[61] Chitturi S Farrell GC. Etiopathogenesis of Nonalcoholic Steatohepatitis. Semin Liver Dis (2001) 21:27–41. doi: 10.1055/s-2001-12927 11296694

[62] Kudo H Takahara T Yata Y Kawai K Zhang W Sugiyama T. Lipopolysaccharide Triggered TNF-Alpha-Induced Hepatocyte Apoptosis in a Murine Non-Alcoholic Steatohepatitis Model. J Hepatol (2009) 51:168–75. doi: 10.1016/j.jhep.2009.02.032 19446916

[63] Verdam FJ Rensen SS Driessen A Greve JW Buurman WA. Novel Evidence for Chronic Exposure to Endotoxin in Human Nonalcoholic Steatohepatitis. J Clin Gastroenterol (2011) 45:149–52. doi: 10.1097/MCG.0b013e3181e12c24 20661154

[64] Loguercio C De Simone T D’Auria MV de Sio I Federico A Tuccillo C. Italian AISF Clinical Group. Non-Alcoholic Fatty Liver Disease: A Multicentre Clinical Study by the Italian Association for the Study of the Liver. Dig Liver Dis (2004) 36:398–405. doi: 10.1016/S1590-8658(04)00094-5 15248380

[65] Ansell J Widrich W Johnson W Fine J. Endotoxin and Bacteria in Portal Blood. Gastroenterology (1977) 73:1190. doi: 10.1016/S0016-5085(19)31902-X 908506

[66] Imajo K Fujita K Yoneda M Nozaki Y Ogawa Y Shinohara Y. Hyperresponsivity to Low-Dose Endotoxin During Progression to Nonalcoholic Steatohepatitis Is Regulated by Leptin-Mediated Signaling. Cell Metab (2012) 16:44–54. doi: 10.1016/j.cmet.2012.05.012 22768838

[67] Huang H Liu T Rose JL Stevens RL Hoyt DG. Sensitivity of Mice to Lipopolysaccharide Is Increased by a High Saturated Fat and Cholesterol Diet. J Inflammation (Lond) (2007) 4:22. doi: 10.1186/1476-9255-4-22 PMC2186306 17997851

[68] Roncon-Albuquerque R Jr Moreira-Rodrigues M Faria B Ferreira AP Cerquiera C Lourenço AP. Attenuation of the Cardiovascular and Metabolic Complications of Obesity in CD14 Knockout Mice. Life Sci (2008) 83:502–10. doi: 10.1016/j.lfs.2008.07.021 18761356

[69] Delude RL Savedra R Jr. Zhao H Thieringer R Yamamoto S Fenton MJ. CD14 Enhances Cellular Responses to Endotoxin Without Imparting Ligand-Specific Recognition. Proc Natl Acad Sci USA (1995) 92:9288–92. doi: 10.1073/pnas.92.20.9288 PMC40970 7568119

[70] Ferrero E Jiao D Tsuberi BZ Tesio L Rong GW Haziot A. Transgenic Mice Expressing Human CD14 Are Hypersensitive to Lipopolysaccharide. Proc Natl Acad Sci USA (1993) 90:2380–4. doi: 10.1073/pnas.90.6.2380 PMC46090 7681594

[71] Haziot A Ferrero E Lin XY Stewart CL Goyert SM. CD14-Deficient Mice Are Exquisitely Insensitive to the Effects of LPS. Prog Clin Biol Res (1995) 392:349–51. doi: 10.1073/pnas.90.6.2380 8524940

[72] Su GL. Lipopolysaccharides in Liver Injury: Molecular Mechanisms of Kupffer Cell Activation. Am J Physiol Gastrointest Liver Physiol (2002) 283:G256–65. doi: 10.1152/ajpgi.00550.2001 12121871

[73] Wright SD Ramos RA Tobias PS Ulevitch RJ Mathison JC. CD14, A Receptor for Complexes of Lipopolysaccharide (LPS) and LPS Binding Protein. Science (1990) 249:1431–3. doi: 10.1126/science.1698311 1698311

[74] Brun P Castagliuolo I Floreani AR Buda A Palù G Martines D. Increased Risk of NASH in Patients Carrying the C(-159)T Polymorphism in the CD14 Gene Promoter Region. Gut (2006) 55:1212. doi: 10.1136/gut.2006.093336 PMC1856285 16849359

[75] Considine RV Sinha MK Heiman ML Kriauciunas A Stephens TW Nyce MR. Serum Immunoreactive-Leptin Concentrations in Normal-Weight and Obese Humans. N Engl J Med (1996) 334:292–5. doi: 10.1056/NEJM199602013340503 8532024

[76] Halaas JL Gajiwala KS Maffei M Cohen SL Chait BT Rabinoqitz D. Weight-Reducing Effects of the Plasma Protein Encoded by the Obese Gene. Science (1995) 269:543–6. doi: 10.1126/science.7624777 7624777

[77] Kessoku T Imajo K Honda Y Kato T Ogawa Y Tomeno W. Resveratrol Ameliorates Fibrosis and Inflammation in a Mouse Model of Nonalcoholic Steatohepatitis. Sci Rep (2016) 6:22251. doi: 10.1038/srep22251 26911834 PMC4766502

[78] Bailey MA Holscher HD. Microbiome-Mediated Effects of the Mediterranean Diet on Inflammation. Adv Nutr (2018) 9:193–206. doi: 10.1093/advances/nmy013 29767701 PMC5952955

[79] Nymark M Pussinen PJ Tuomainen AM Forsblom C Groop P Lehto M. Serum Lipopolysaccharide Activity Is Associated With the Progression of Kidney Disease in Finnish Patients With Type 1 Diabetes. Diabetes Care (2009) 32:1689–93. doi: 10.2337/dc09-0467 PMC2732155 19502539

[80] Umoh FI Kato I Ren J Wachowiak PL Ruffin 4MT Turgeon DK. Markers of Systemic Exposures to Products of Intestinal Bacteria in a Dietary Intervention Study. Eur J Nutr (2016) 55:793–8. doi: 10.1007/s00394-015-0900-7 PMC4619169 25903259

[81] Mani V Hollis JH Gabler NK. Dietary Oil Composition Differentially Modulates Intestinal Endotoxin Transport and Postprandial Endotoxemia. Nutr Metab (Lond) (2013) 10:6. doi: 10.1186/1743-7075-10-6 23305038 PMC3577458

[82] Lyte JM Gabler NK Hollis JH. Postprandial Serum Endotoxin in Healthy Humans Is Modulated by Dietary Fat in a Randomized, Controlled, Cross-Over Study. Lipids Health Dis (2016) 15:186. doi: 10.1186/s12944-016-0357-6 27816052 PMC5097840

[83] Erridge C Attina T Spickett CM Webb DJ. A High-Fat Meal Induces Low-Grade Endotoxemia: Evidence of a Novel Mechanism of Postprandial Inflammation. Am J Clin Nutr (2007) 86:1286–92. doi: 10.1093/ajcn/86.5.1286 17991637

[84] Laugerette F Vors C Peretti N Michalski M. Complex Links Between Dietary Lipids, Endogenous Endotoxins and Metabolic Inflammation. Biochimie (2011) 93:39–45. doi: 10.1016/j.biochi.2010.04.016 20433893

[85] Xue L He J Gao N Lu X Li M Wu X. Probiotics May Delay the Progression of Nonalcoholic Fatty Liver Disease by Restoring the Gut Microbiota Structure and Improving Intestinal Endotoxemia. Sci Rep (2017) 7:45176. doi: 10.1038/srep45176 28349964 PMC5368635

[86] Eslamparast T Eghtesad S Hekmatdoost A Poutschi H. Probiotics and Nonalcoholic Fatty Liver Disease. Middle East J Dig Dis (2013) 5:129–36. PMC3990183 24829682

[87] Han C Ding Z Shi H Qian W Hou Q Lin R. The Role of Probiotics in Lipopolysaccharide-Induced Autophagy in Intestinal Epithelial Cells. Cell Physiol Biochem (2016) 38:2464–78. doi: 10.1159/000445597 27309845

[88] Hu H Lin A Kong M Yao X Yin M Xia H. Intestinal Microbiome and NAFLD: Molecular Insights and Therapeutic Perspectives. J Gastroenterol (2020) 55:142–58. doi: 10.1007/s00535-019-01649-8 PMC6981320 31845054

[89] Scarpignato C Pelosini I. Rifaximin, A Poorly Absorbed Antibiotic: Pharmacology and Clinical Potential. Chemotherapy (2005) 51:36–66. doi: 10.1159/000081990 15855748

[90] Gangarapu V Ince AT Baysal B Kayar Y Kiliç U Gök Ö. Efficacy of Rifaximin on Circulating Endotoxins and Cytokines in Patients With Nonalcoholic Fatty Liver Disease. Eur J Gastroenterol Hepatol (2015) 27:840–5. doi: 10.1097/MEG.0000000000000348 26043290

[91] Abdel-Razik A Mousa N Shabana W Refaey M Elzehery R Elhelaly R. Rifaximin in Nonalcoholic Fatty Liver Disease: Hit Multiple Targets With a Single Shot. Eur J Gastroenterol Hepatol (2018) 30:1237–46. doi: 10.1097/MEG.0000000000001232 30096092

[92] Ponziani FR Zocco MA D’Aversa F Pompili M Gasbarrini A. Eubiotic Properties of Rifaximin: Disruption of the Traditional Concepts in Gut Microbiota Modulation. World J Gastroenterol (2017) 23:4491–9. doi: 10.3748/wjg.v23.i25.4491 PMC5504364 28740337

[93] Darkoh C Lichtenberger LM Ajami N Dial EJ Jiang Z DuPont HL. Bile Acids Improve the Antimicrobial Effect of Rifaximin. Antimicrob Agents Chemother (2010) 54:3618–24. doi: 10.1128/AAC.00161-10 PMC2934964 20547807

[94] Kim MS Morales W Hani AA Kim S Kim G Weitsman S. The Effect of Rifaximin on Gut Flora and Staphylococcus Resistance. Dig Dis Sci (2013) 58:1676–82. doi: 10.1007/s10620-013-2675-0 23589147

[95] Mencarelli A Migliorati M Barbanti M Cipriani S Palladino G Distrutti E Renga B. Pregnane-X-Receptor Mediates the Anti-Inflammatory Activities of Rifaximin on Detoxification Pathways in Intestinal Epithelial Cells. Biochem Pharmacol (2010) 80:1700–7. doi: 10.1016/j.bcp.2010.08.022 20816942

[96] Hirota SA. Understanding the Molecular Mechanisms of Rifaximin in the Treatment of Gastrointestinal Disorders–A Focus on the Modulation of Host Tissue Function. Mini Rev Med Chem (2015) 16:206–17. doi: 10.2174/1389557515666150722105705 26202186

[97] Zhu L Baker RD Baker SS. Gut Microbiome and Nonalcoholic Fatty Liver Diseases. Pediatr Res (2015) 77:245–51. doi: 10.1038/pr.2014.157 25310763

[98] Loguercio C Federico A Tuccillo C Terracciano F D’Auria MV De Simone C. Beneficial Effects of a Probiotic VSL#3 on Parameters of Liver Dysfunction in Chronic Liver Diseases. J Clin Gastroenterol (2005) 39:540–3. doi: 10.1097/01.mcg.0000165671.25272.0f 15942443

[99] Malaguarnera M Vacante M Antic T Giordano M Chisari G Acquaviva R. Bifidobacterium Longum With Fructo-Oligosaccharides in Patients With Non Alcoholic Steatohepatitis. Dig Dis Sci (2012) 57:545–53. doi: 10.1007/s10620-011-1887-4 21901256

[100] Wong VW Tse CH Lam TT Wong WG Chim AM Chu WC. Molecular Characterization of the Fecal Microbiota in Patients With Nonalcoholic Steatohepatitis–a Longitudinal Study. PloS One (2013) 8:e62885. doi: 10.1371/journal.pone.0062885 23638162 PMC3636208

[101] Su D Nie Y Zhu A Chen Z Wu P Zhang L. Vitamin D Signaling Through Induction of Paneth Cell Defensins Maintains Gut Microbiota and Improves Metabolic Disorders and Hepatic Steatosis in Animal Models. Front Physiol (2016) 7:498. doi: 10.3389/fphys.2016.00498 27895587 PMC5108805

[102] Luger M Kruschitz R Kienbacher C Traussnigg S Langer FB Schindler K. Prevalence of Liver Fibrosis and Its Association With Non-Invasive Fibrosis and Metabolic Markers in Morbidly Obese Patients With Vitamin D Deficiency. Obes Surg (2016) 26:2425–32. doi: 10.1007/s11695-016-2123-2 PMC5018030 26989059

[103] Cani PD Possemiers S Van de Wiele T Guiot Y Everard A Rottier O. Changes in Gut Microbiota Control Inflammation in Obese Mice Through a Mechanism Involving GLP-2-Driven Improvement of Gut Permeability. Gut (2009) 58:1091–103. doi: 10.1136/gut.2008.165886 PMC2702831 19240062

[104] Parnell JA Reimer RA. Prebiotic Fibres Dose-Dependently Increase Satiety Hormones and Alter Bacteroidetes and Firmicutes in Lean and Obese JCR: LA-Cp Rats. Br J Nutr (2012) 107:601–13. doi: 10.1017/S0007114511003163 PMC3827017 21767445

[105] Parnell JA Reimer RA. Weight Loss During Oligofructose Supplementation Is Associated With Decreased Ghrelin and Increased Peptide YY in Overweight and Obese Adults. Am J Clin Nutr (2009) 89:1751–9. doi: 10.3945/ajcn.2009.27465 PMC3827013 19386741

[106] Loman BR Hernandez-Saavedra D An R Rector RS. Prebiotic and Probiotic Treatment of Nonalcoholic Fatty Liver Disease: A Systematic Review and Meta-Analysis. Nutr Rev (2018) 76:822–39. doi: 10.1093/nutrit/nuy031 30113661

[107] Ting WJ Kuo WW Hsieh DJ Yeh Y Day C Chen Y. Heat Killed Lactobacillus Reuteri GMNL-263 Reduces Fibrosis Effects on the Liver and Heart in High Fat Diet-Hamsters *via* TGF-Beta Suppression. Int J Mol Sci (2015) 16:25881–96. doi: 10.3390/ijms161025881 PMC4632831 26516851

[108] Hsieh FC Lee CL Chai CY Chen WT Lu YC Wu CS. Oral Administration of Lactobacillus Reuteri GMNL-263 Improves Insulin Resistance and Ameliorates Hepatic Steatosis in High Fructose-Fed Rats. Nutr Metab (Lond) (2013) 10:35. doi: 10.1186/1743-7075-10-35 23590862 PMC3637306

[109] Uchinaka A Azuma N Mizumoto H Nakano S Minamiya M Yoneda M. Anti-Inflammatory Effects of Heat-Killed Lactobacillus Plantarum L-137 on Cardiac and Adipose Tissue in Rats With Metabolic Syndrome. Sci Rep (2018) 8:8156. doi: 10.1038/s41598-018-26588-x 29802339 PMC5970162

[110] Izumo T Ida M Maekawa T Furukawa Y Kitagawa Y Kiso Y. Comparison of the Immunomodulatory Effects of Live and Heat-Killed Lactobacillus Pentosus S-Pt84. J Health Sci (2011) 57:304–10. doi: 10.1248/jhs.57.304

[111] Nonaka Y Izumo T Izumi F Maekawa T Shibata H Nakano A. Antiallergic Effects of Lactobacillus Pentosus Strain S-PT84 Mediated by Modulation of Th1/Th2 Immunobalance and Induction of IL-10 Production. Int Arch Allergy Immunol (2008) 145:249–57. doi: 10.1159/000109294 17914277

[112] Sakai Y Arie H Ni Y Zhuge F Xu L Chen G. Lactobacillus Pentosus Strain S-PT84 Improves Steatohepatitis by Maintaining Gut Permeability. J Endocrinol (2020) 247:169–81. doi: 10.1530/JOE-20-0105 33032263

[113] Zhao X Higashikawa F Noda M Kawamura Y Matoba Y Kumagai T. The Obesity and Fatty Liver Are Reduced by Plant-Derived Pediococcus Pentosaceus LP28 in High Fat Diet-Induced Obese Mice. PloS One (2012) 7:e30696. doi: 10.1371/journal.pone.0030696 22363472 PMC3281851

[114] Fei G Raehal K Liu S Qu M Sun X Wang G. Lubiprostone Reverses the Inhibitory Action of Morphine on Intestinal Secretion in Guinea Pig and Mouse. J Pharmacol Exp Ther (2010) 334:333–40. doi: 10.1124/jpet.110.166116 PMC2912047 20406855

[115] Hayashi S Kurata N Yamaguchi A Amagase K Takeuchi K. Lubiprostone Prevents Nonsteroidal Anti-Inflammatory Drug-Induced Small Intestinal Damage by Suppressing the Expression of Inflammatory Mediators *via* EP4 Receptors. J Pharmacol Exp Ther (2014) 349:470–9. doi: 10.1124/jpet.114.213991 24713141

[116] Arakawa K Ishigami T Nakai-Sugiyama M Chen L Doi H Kino T. Lubiprostone as a Potential Therapeutic Agent to Improve Intestinal Permeability and Prevent the Development of Atherosclerosis in Apolipoprotein E-Deficient Mice. PloS One (2019) 14:e0218096. doi: 10.1371/journal.pone.0218096 31206525 PMC6576757

[117] Nighot PK Leung L Ma TY. Chloride Channel ClC- 2 Enhances Intestinal Epithelial Tight Junction Barrier Function *via* Regulation of Caveolin-1 and Caveolar Trafficking of Occludin. Exp Cell Res (2017) 352:113–22. doi: 10.1016/j.yexcr.2017.01.024 PMC5328798 28161538

